# Effect of Hyaluronan on Developmental Competence and Quality of Oocytes and Obtained Blastocysts from *In Vitro* Maturation of Bovine Oocytes

**DOI:** 10.1155/2014/519189

**Published:** 2014-02-13

**Authors:** Jolanta Opiela, Joanna Romanek, Daniel Lipiński, Zdzisław Smorąg

**Affiliations:** ^1^Department of Biotechnology of Animal Reproduction, National Research Institute of Animal Production, Krakowska 1 Street, 32-083 Balice, Kraków, Poland; ^2^Department of Biochemistry and Biotechnology, Poznań University of Life Sciences, Dojazd 11 Street, 60-632 Poznań, Poland; ^3^Institute of Human Genetics, Polish Academy of Sciences, Strzeszyńska 32, 60-479 Poznań, Poland

## Abstract

The objective of the present study was to evaluate the effect of hyaluronan (HA) during IVM on meiotic maturation, embryonic development, and the quality of oocytes, granulosa cells (GC), and obtained blastocysts. COCs were matured *in vitro* in control medium and medium with additional 0.035% or 0.07% of exogenous HA. The meiotic maturity did not differ between the analysed groups. The best rate and the highest quality of obtained blastocysts were observed when 0.07% HA was used. A highly significant difference (P < 0.001) was noted in the mean number of apoptotic nuclei per blastocyst and in the DCI between the 0.07% HA and the control blastocysts (P < 0.01). Our results suggest that addition of 0.035% HA and 0.07% HA to oocyte maturation media does not affect oocyte nuclear maturation and DNA fragmentation. However, the addition of 0.07% HA during IVM decreases the level of blastocysts DNA fragmentation. Finally, our results suggest that it may be risky to increase the HA concentration during IVM above 0.07% as we found significantly higher *Bax* mRNA expression levels in GC cultured with 0.07% HA. The final concentration of HA being supplemented to oocyte maturation media is critical for the success of the IVP procedure.

## 1. Introduction

The large discrepancy between the number of oocytes undergoing *in vitro* fertilisation and the number of embryos developing to the blastocyst stage (approximately 30–40%) calls for further research to improve the oocyte maturation medium in order to mimic the in vivo maturation conditions. A few studies have focused their attention on the supplementation of *in vitro* embryo culture media with hyaluronan (HA) due to its biochemical properties [1–3]. Important biological functions are not properly regulated during *in vitro* culture due to the two-dimensional nature of the culture. Hyaluronan forms a concentration dependent, three-dimensional, jelly-like network. A three-dimensional network improves the interaction between cellular receptors and the molecules in the external environment by improving the binding of molecules such as polypeptide growth factors that play an important role during early embryonic development [4]. Hyaluronan is a major component of the cumulus-oocyte complex (COCs) and is synthesised and secreted by granulosa cells under the stimulation of LH and FSH [5]. The insufficient interactions of hyaluronan and its receptor CD44 during *in vitro* maturation may decrease the capacity of fertilization and development of oocytes matured *in vitro* [6]. The resumption of meiosis in mares, pigs, and rats is associated with decreased levels of connexin 43 (Cx43) [7]. Yokoo et al. [8] revealed that the interaction of HA with the CD44 receptor in granulosa cells during cumulus expansion lowers the levels of Cx43. As a result of this process, the transport of cAMP between cells and the oocyte is blocked. A decrease in the concentration of cAMP in oocytes is a signal for the activation of MPF, which is responsible for the resumption of meiosis (GVBD) [8]. In addition, HA participates in the process of sperm capacitation [9], determines the proper course of fertilisation, and affects the growth potential of early embryos [10]. Many cellular processes are regulated by the interaction between HA and its surface receptors, such as CD44, RHAMM (receptor for HA mediated motility), and ICAM-1 (intercellular adhesion molecule 1) [11, 12]. These processes include the maintenance of tissue homeostasis [13], cell adhesion and migration [14, 15], cell proliferation and differentiation, and the inhibition of apoptosis and immune response [16, 17].

The objective of the present study was to evaluate the HA supplementation effect on meiotic maturation at two concentrations within *in vitro* maturation medium. Additionally, embryonic development after *in vitro* fertilisation as well as the quality of bovine oocytes, granulosa cells that served as coculture during IVM, and obtained blastocysts will be evaluated. The results of our previous experiments [18] have indicated that the supplementation of IVM with HA did not increase the nuclear maturity of oocytes. Moreover, our previous results have indicated that the 0.035% and 0.07% HA concentrations present opposing effects on IVM. The meiotic maturity of the 0.07% HA treated oocytes was the lowest [18], inclining us to apply them in this experiment. Therefore, in the present experiment, we TUNEL analysed oocytes in two experimental groups. Moreover, blastocysts obtained after fertilisation of HA treated COCs provided data on their developmental competence.

## 2. Materials and Methods

Immature oocytes were isolated from ovaries obtained from heifers and cows from the slaughterhouse, which were delivered in a warm flask to the laboratory within 2-3 hours after slaughter.

### 2.1. Chemicals and Supplies

The reagents used in the presented experiments were purchased from Sigma-Aldrich, Poland, unless otherwise indicated.

### 2.2. Experimental Design

The research was performed in two directions. First, we evaluated the effect of two concentrations of HA supplementation on meiotic maturation and embryonic development after *in vitro* fertilisation. Second, we evaluated the possible negative impact of HA on the oocytes, the blastocysts, and granulosa cells. TUNEL staining and the estimation of apoptotic genes transcripts expression by real-time PCR were employed as quality markers.

### 2.3. Recovery and Selection of Bovine Oocytes

The detailed description of procedure is provided in Opiela et al. [18]. Cumulus-oocyte complexes (COCs) were retrieved from ovaries of slaughtered cows by aspiration of follicular fluid of ovarian follicles. For this purpose, a syringe and needle were used. Follicular fluid was collected into tubes (Corning, 12 mL) and left for 15–30 minutes at room temperature to sediment. The supernatant was then collected, and the remaining sediment was transferred into a holding medium (TCM 199—Earle's salt with 25 mM of HEPES containing 10% foetal calf serum). Next, COCs were recovered under stereomicroscope. Before placing COCs into the *in vitro* maturation, they were washed three times for selection. Degenerated oocytes and those without compact cumulus cells were discarded.

### 2.4. *In Vitro* Maturation in Coculture with Granulosa Cells

For the culture of COCs the commercial medium TCM-199 buffered with sodium bicarbonate and supplemented with L-glutamine was used. Selected COCs were cultured for 22 hours in 2 mL of medium supplemented with 20% estrus cow serum (ECS, heat inactivated-prepared in our laboratory) and an additional 3 to 5 × 10^6^ granulosa cells/mL (GC). Between 30 and 40 COCs were used per culture dish. Granulosa cells were obtained from follicles with a diameter of 4–6 mm without atretic changes and containing oocytes with normal morphological appearance. The obtained granulosa cells were suspended in 7 mL of *in vitro* maturation medium without exogenous HA. After carefully mixing the medium with GC, the suspension was divided between 3 culture dishes, for 2 mL each. *In vitro* maturation was performed with different concentrations of 1% high molecular weight HA (Croma Pharma GmbH, Austria), with 75 *μ*L/2 mL medium (a final concentration of HA 0.035%) and 150 *μ*L/2 mL medium (a final concentration of 0.07%). As a control, we used COCs matured only in maturation medium and cocultured with granulosa cells [18]. This approach was provided the same way to experimental conditions in all of the groups, as the estrus cow serum may contain HA and HA is also synthesised and secreted by granulosa cells in undefined and variable amount. Moreover, the same ESC was used in all IVM media during the whole experiment and each repetition of IVM/IVF/IVC was performed in three simultaneous experimental groups. Oocytes matured for 22 h at 38.5°C under 5% CO_2_ in air.

### 2.5. Effect of HA on Meiotic Maturity and Chromatin Fragmentation in Oocytes

#### 2.5.1. TUNEL

The detailed description of procedure is provided in Opiela et al. [18]. TUNEL analysis was performed using a Deadend Fluorometric TUNEL System (Promega, Poland).

Fixed denuded oocytes were first incubated with 0.2% Triton X-100 solution for 5 minutes, then a reaction mixture consisting of equilibration buffer, the mixture of nucleotides and TdT enzyme for 1 h at maximum humidity, and lastly a solution of 2 × SSC for 15 mins. After the performed incubations, the oocytes were washed three times in PBS/PVP for at least 5 minutes. Finally, the oocytes were placed in a drop of VECTASHIELD + DAPI solution. Oocytes that were treated with UV light for 30 mins immediately after maturation were used as a positive control. As a negative control, oocytes placed in the reaction mixture lacking the rTdT enzyme were used. Oocytes were analysed under a fluorescent microscope. To visualise the stages of meiosis and DNA fragmentation of oocytes, a filter of >460 nm was used for the blue fluorescence of DAPI stained cells and a filter with a wavelength of 520 ± 20 nm was used for the green fluorescence of apoptotic cells, which incorporated into the nucleus with a fluorescein-conjugated dUTP.

#### 2.5.2. Estimation of Meiotic Maturity

We have applied similar criteria to those of Warzych et al. [19]. In cases of strong fluorescent/DAPI signals from the nuclei and the polar body, meiotic stage classification (GV, MI, AI, TI, MII) was performed. A detailed description of assessment criteria is also available in Opiela et al. [18]. Briefly, the germinal vesicle (GV) was characterised by varying degrees of chromatin condensation, metaphase of first meiotic division (MI) was characterised by chromosomes arranged as a group of separated bivalents, and metaphase second (MII) was characterised by the presence of haploid set of chromosomes and chromosomes forming the first polar body (PB1). Meiotic maturity was counted as the ratio of oocytes in metaphase II stage to the total number of analysed oocytes.

#### 2.5.3. Estimation of Oocytes Apoptotic Index DCI

TUNEL positive oocytes indicated fragmented DNA by a strong fluorescent/FITC signal from the nuclei or polar body. As a control for the experiment, the COCs were subjected to the UV (30 mins. at room temperature in the M199 medium containing HEPES supplemented with 5% FCS). This generated “damage” to the DNA, which would show a positive TUNEL signal and indicate that the TUNEL reaction works. The death cell index (DCI) was expressed as a ratio of apoptotic oocytes exhibiting a strong fluorescent/FITC signal to all analysed oocytes from each group [18, 19].

### 2.6. Effect of HA on the Developmental Competence of Bovine Oocytes

#### 2.6.1. *In Vitro* Fertilisation

IVF was performed according to our standard protocol that was previously described [20, 21]. Briefly, after 22–24 hrs of maturation, the selected oocytes were fertilised using frozen semen from single bull. The motile sperm were selected by centrifugation (300 g at room temp.) on a discontinuous (1 mL 45% over 1 mL 90%) Percoll (Pharmacia, Sweden). After washing, the sperm were suspended in the fertilisation medium containing 10 *μ*g/mL of heparin and a mixture of penicillamine (20 *μ*M), hypotaurine (10 *μ*M), and epinephrine (1 *μ*M) at a concentration of 1-2 × 10^6^ spermatozoa/mL of medium. After washing and partial deprivation of the expanded cumulus, the mature COCs were transferred in groups of 10 into 50 *μ*L of fertilisation medium. Gametes were incubated together for 18 to 21 h at 38.5°C under 5% CO_2_ in air [20, 21].

#### 2.6.2. *In Vitro* Embryo Culture and VERO Cell Preparation

IVC and VERO cell preparation was performed according to our previously described standard protocol [20, 21]. Briefly, at 24 h after fertilisation, the presumptive zygotes were transferred into 50 *μ*L drops of B_2_ medium (C.C.D., Paris, France) under mineral oil. The number of cleaved embryos was recorded on Day 2 following fertilisation, and approximately 20 embryos were placed in coculture with Vero cells in 50 *μ*L drops of B_2_ medium supplemented with 2.5% FCS, under mineral oil. Medium in culture drops was partially changed (20 *μ*L) at intervals of 48 h. Embryos were maintained in coculture for 7 to 8 days. At the end of the culture period, the total blastocyst rate and the hatching rate were recorded.

Vero cells were obtained frozen from ECAGC, Salisbury, UK. Cells were seeded at a concentration of 1 × 10^6^ cells in 5 mL of medium per flask (for passages) and 1 × 10^2^ cells in 50 *μ*L of medium per drop (for coculture with the small group of embryos). The medium for coculture, B_2_ medium enriched with 2.5% FCS, was changed before the embryos were added and then partially changed every 48 h. The *in vitro* embryo culture was performed at 38.5°C under 5% CO_2_ in air.

### 2.7. Effect of HA on Blastocysts Quality Measured by TUNEL

TUNEL analysis was performed using a Deadend Fluorometric TUNEL System, (Promega, Poland) according to the protocol described in Warzych et al. [19]. The analysis was performed using a fluorescent microscope (Nikon Eclipse E600).

#### 2.7.1. Estimation of Blastocysts Apoptotic Index, DCI

Day 8 blastocysts (middle, late, expanding, and hatched) were subjected to TUNEL analysis. The number of all blastomere nuclei and the number of all apoptotic nuclei were recorded for each embryo. To assess the DCI for the single blastocyst the sum of all apoptotic nuclei detected in the analysed blastocyst was divided by the sum of all nuclei detected in analysed blastocyst and multiplied by 100.

### 2.8. Effect of HA on *Bax* and *Bcl-2* Transcript Levels in Granulosa Cells Used as Coculture during IVM

#### 2.8.1. RNA Isolation, RT, and Quantitative PCR (qPCR)

After IVM, the remaining culture medium of the three experimental groups with cocultured granulosa cells was transferred into Eppendorf tubes for centrifugation (300 ×g). The remaining pellet of cells was washed in PBS (without Mg^2+^ and Ca^2+^) three times. After washing, the cells were resuspended in 10 *μ*L of PBS, snap frozen in liquid nitrogen, and stored at −80°C until use. The samples from the three experimental groups had the same number of cells as during the granulose-cells coculture setup. The cells obtained from the follicles were equally divided into three culture dishes, the control, HA at 0.035%, and HA at 0.07%. This experiment was repeated three times with the same COCs number for each repetition (40–50 COCs).

Total RNA was isolated as previously described [22]. The relative expression levels of glyceraldehyde-3-phosphate dehydrogenase (GAPDH) were used to normalise the marker gene expression in each sample. A One-Step Brilliant II SYBR Green QRT-PCR Master Mix Kit (Stratagene, La Jolla, CA, USA) was used to perform relative quantification of gene expression. Each PCR reaction (total volume of 25 *μ*L) consisted of total RNA (2 ng/*μ*L), 1× of SYBR Green QRT-PCR master mix (contains an optimised RT-PCR buffer, 2.5 nM of MgCl2, nucleotides (GAUC), SureStart Taq DNA polymerase, SYBR Green and stabilisers), 200 nM each of the forward and reverse primer [21], and 1× of RT/RNase block enzyme mixture. Thermal cycling conditions were as follows: 30 mins at 50°C (for the first-strand synthesis); 10 mins at 95°C; 35 cycles of 30 s at 95°C for denaturing; 60 s at 57°C for annealing; and 30 s at 72°C for extension. Experiments were carried out on Mastercycler ep realplex apparatus (Eppendorf, UK Limited, Cambridge). GAPDH was used as an endogenous standard. The results for individual target genes were normalised according to the relative concentration of the endogenous standard. Each reaction was run in triplicate and the obtained results were averaged. The results for the GC HA 0.035% and GC HA 0.07% were compared with control granulosa cells which served as a calibrator. The 2–[delta][delta]Ct method was used for calculating the relative quantification.

### 2.9. Statistical Analysis

Differences in TUNEL results from oocytes and developmental competence of fertilised oocytes were assessed using chi-square test (*χ* ^2^). Differences in *Bax* and *Bcl-2* transcripts level were assessed using ANOVA followed by Tuckey's post hoc test. The same test was performed in case of TUNEL results from blastocysts regarding all nuclei, apoptotic nuclei, and DCI. In all tests differences with a probability value of 0.05 or less were considered significant.

## 3. Results

### 3.1. Effect of HA on the Meiotic Maturity and Chromatin Fragmentation of Oocytes

**Figure 1:**
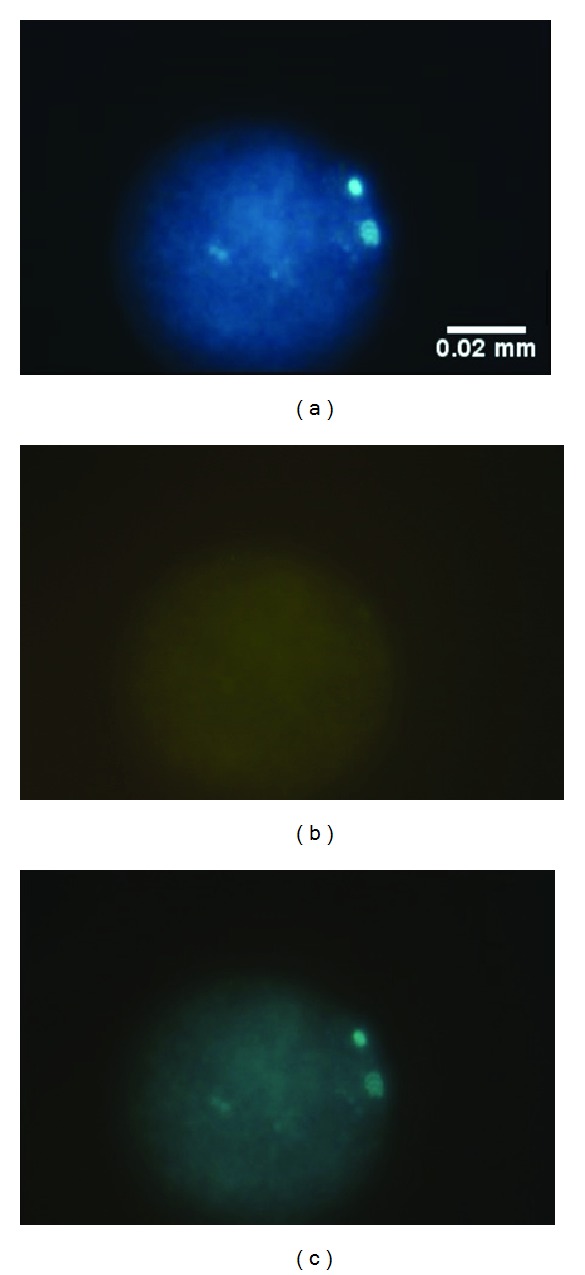
Bovine oocyte after TUNEL staining. (a) DAPI staining of chromatin in metaphase II stage and the 1st polar body; (b) absence of green fluorescence after FITC staining of the same oocyte; (c) photos (a) and (b) merged.

To evaluate the HA impact on oocytes after *in vitro* culture, the estimation of meiosis stage and the level of DNA fragmentation was performed by TUNEL staining (Figure 1). The TUNEL staining was analysed from 428 oocytes matured under three media conditions in a volume of 2 mL in the presence of granulosa cells. Of the oocytes evaluated, 151 oocytes in the control medium without HA matured, 133 of analysed oocytes matured in medium with 0.035% HA, and 144 oocytes matured in medium with 0.07% HA. The highest meiotic maturation (70.2%) was observed in oocytes cultured in the control medium, and the lowest maturation was in medium supplemented with 0.07% HA (63.1%) (Table 1). In medium supplemented with 0.035% HA, the meiotic maturity was almost 67.7% (Table 1). The obtained meiotic maturity did not differ between the analysed groups. We did not find any signs of DNA fragmentation in any of oocytes within the three analysed groups (Figure 1). This experiment showed that HA supplementation did not have a detrimental impact on oocyte chromatin integrity.

### 3.2. Effect of HA on the Developmental Competence of Bovine Oocytes

Although the number of cleaved eggs was significantly lower when gametes were matured in the presence of HA (66% and 73% for 0.035 and 0.07% HA, resp.), the number of obtained blastocysts was the highest when 0.07% HA was used. However, there was no significant difference between this group of oocytes and control (Table 2). The lowest number of cleaved eggs was observed for oocytes matured with 0.035% HA in relation to the control (*P* < 0.001) and medium supplemented with 0.07% HA (*P* < 0.05). The lowest number of obtained blastocysts was observed for oocytes matured with 0.035% HA relative to the medium with 0.07% HA (*P* < 0.05; Table 2).

### 3.3. Effect of HA on Blastocysts Quality Measured by TUNEL

**Figure 2:**
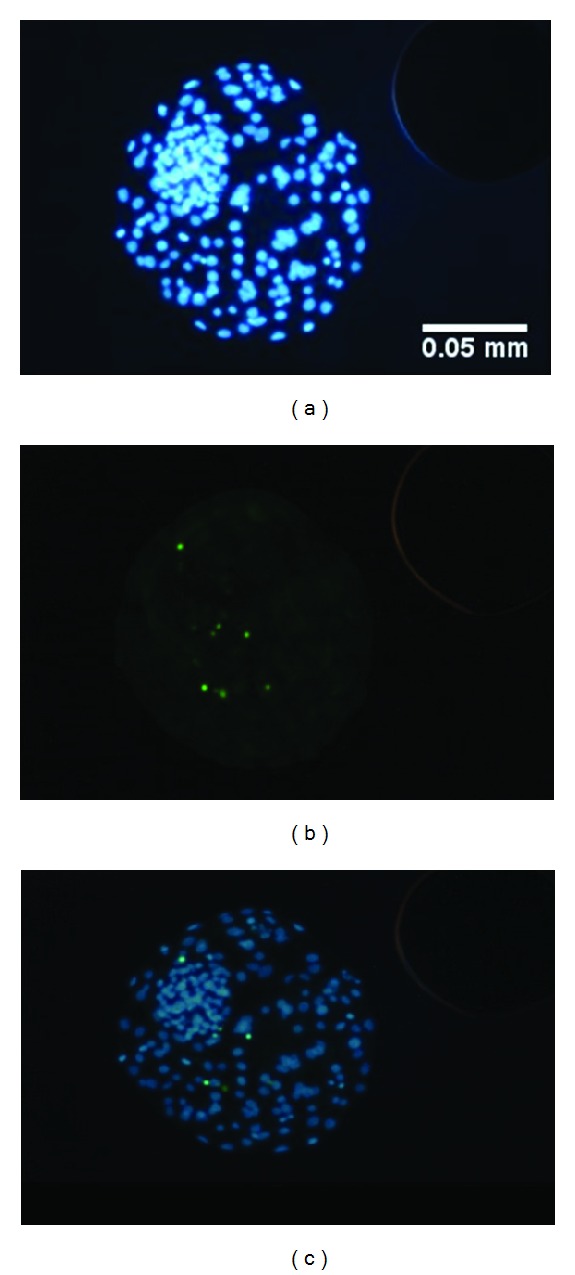
Bovine blastocyst after TUNEL staining. (a) DAPI staining of all blastomere nuclei; (b) green FITC staining of apoptotic nuclei; (c) photos (a) and (b) merged.

The quality of blastocysts developed from oocytes matured with 0.07% HA was the highest. A highly significant difference (*P* < 0.001) was noted in the mean number of apoptotic nuclei per blastocyst and a highly significant difference (*P* < 0.01) was noted in the DCI between the 0.07% HA blastocysts and the control blastocysts (Table 3, Figure 2). The control blastocysts had the highest mean number of apoptotic nuclei per blastocyst when compared to blastocysts developed from oocytes cultured with 0.07% HA (*P* < 0.001) and 0.035% HA (*P* < 0.05) (Table 3). There were no significant differences between the mean number of nuclei per blastocyst in all analysed groups of blastocysts (Table 3, Figure 2).

### 3.4. Effect of HA on *Bax* and *Bcl-2* Transcript Levels in Granulosa Cells

**Figure 3:**
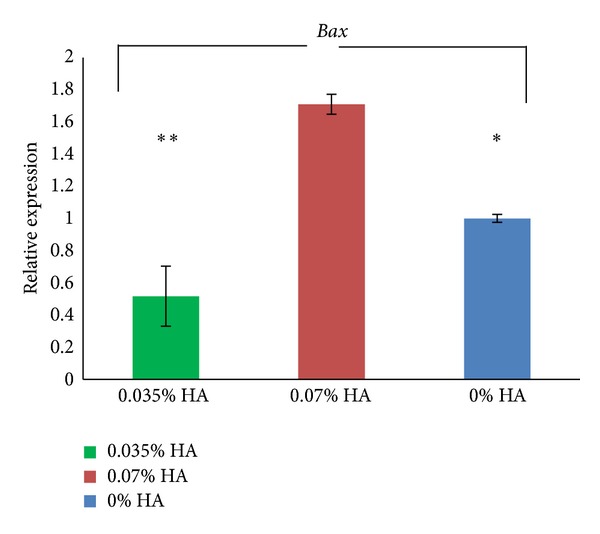
Relative mRNA expressions (mean ± SD) for *Bax* in granulosa cells which served as coculture during IVM of oocytes cultured with addition of 0.035% HA, 0.07% HA, and control. The significant differences were noted **(*P* < 0.01), *(*P* < 0.05) (ANOVA followed by Tuckey's post hoc test)

**Figure 4:**
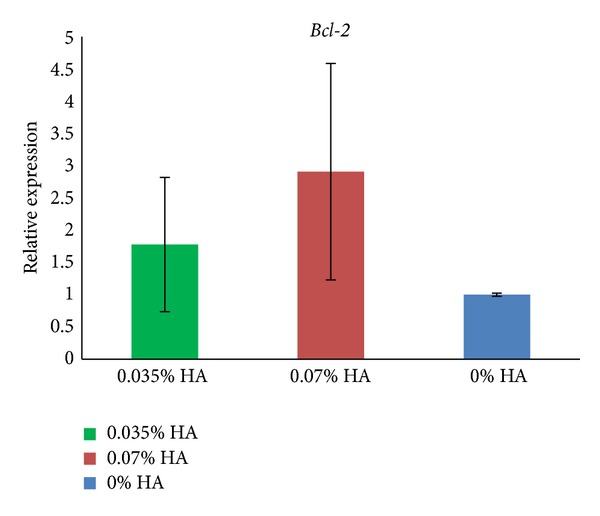
Relative mRNA expressions (mean ± SD) for *Bcl-2* in granulosa cells which served as coculture during IVM of oocytes. The expression of levels of *Bcl-2* in granulosa cells was measured after the addition of 0.035% HA, 0.07% HA, and at control levels. No significant differences were noted (ANOVA followed by Tuckey's post hoc test).

To better analyse the possible negative impact of applied concentrations of HA for IVM conditions, we estimated the relative *Bax* and *Bcl-2* transcript levels in cocultured granulosa cells. We found significantly higher *Bax* mRNA expression in GC cultured with 0.07% HA as compared to the control (*P* < 0.05) and from 0.035% HA GC (*P* < 0.01) (Figure 3). No significant differences were noted in the relative *Bcl-2* expression between the analysed groups of GC (Figure 4).

## 4. Discussion

The present study evaluated the effect of exogenous HA supplementation of *in vitro* maturation medium. Changes in meiotic maturation, embryonic development after *in vitro* fertilisation, the quality of bovine oocytes and IVM cocultured granulosa cells, and the quality of obtained blastocysts were measured. Yokoo et al. [6] showed that after culture of oocytes in the presence of anti-CD44 antibody, the suppression of MPF and an inability to induce GVBD were observed. The interaction between hyaluronan and its main receptor CD44 results in closing the gap junctions and the cessation cAMP transport from granulosa cells to the ooplasm, which in turn triggers the activation of MPF and resumption of meiosis [6]. Based on these data, it was assumed that the addition of exogenous hyaluronan could increase the percentage of oocytes reaching the metaphase II stage. So far, there is only one paper reporting on the role of exogenous HA supplementation during bovine *in vitro* oocyte maturation and further embryo development [23]. The authors matured oocytes in the presence or absence of HA, Hyal-2 (Hyaluronidase enzyme), or 4-methylumbelliferone (4-MU), an HA synthesis inhibitor [23]. When we started our research, no information was available regarding the HA concentration for successful bovine oocyte maturation and blastocyst development after fertilisation. During preparation of this paper, Marei et al. [23] published their paper reporting the effect of IVM supplementation with HA at 0.1, 0.5 and 1 mg/mL on oocyte maturation but only reported the effect of 0.5 mg/mL of HA on embryo development and quality. Their concentration of 0.5 mg/mL is the mean concentration of the 0.75 mg/mL and 0.375 mg/mL applied by us. Marei et al. [23] have shown that the addition of 0.5 mg/mL of HA to oocyte maturation media did not affect cumulus cell expansion or oocyte nuclear maturation.

Our results support their observations, as we did not find any significant difference between the analysed groups. However, based on the rates of mature oocytes in three experimental oocytes groups, it is noticeable that the highest meiotic maturation was observed in oocytes cultured in control medium and the lowest in medium supplemented with 0.07% HA. Based on data regarding HA synthesis and reduction [24, 25], the authors [23] suggested that a low concentration of HA is required for signalling mechanisms contributing to oocyte nuclear maturation and that no effect can be seen on nuclear maturation if HA production is not markedly inhibited. In contrast, high HA concentration could be harmful [26]. Our results seem to support this assumption. We also did not find any signs of DNA fragmentation in any of oocytes within the three analysed groups, which proves that HA does not have a detrimental impact on DNA fragmentation.

Although the number of cleaved eggs was significantly lower when gametes were matured in presence of HA, the number of obtained blastocysts was the highest when 0.07% HA was used. However, there was no significant difference between this group of oocytes and control. These data make determining the effect of HA difficult. Looking at the higher percentage of developed blastocysts in the 0.07% HA group as compared to the remaining two groups, it can be assumed that a significant difference could be noted when larger populations of oocytes are fertilised. Still, it is quite an interesting observation that a statistically lower number of obtained blastocysts developed from oocytes matured in 0.035% HA as compared to 0.07% HA. It seems that the applied HA concentration is the key to success. We assume that there is a certain threshold level of HA after which the positive effect is reached.

This explanation may be confirmed by another observation regarding blastocyst quality as measured by TUNEL staining. Interestingly, we found that blastocysts developed from oocytes matured with 0.07% HA had the best quality. A high significant difference in DCI was noted between these blastocysts and the control (*P* < 0.001). The number of apoptotic nuclei in blastocysts developed from oocytes cultured in the presence of HA (both concentrations) was statistically lower when compared to the control. Based on these results, we can conclude that supplementation of exogenous HA during oocyte IVM improves the quality of developed embryos.

To better analyse the possible negative impact of applied concentrations of HA for IVM conditions, we estimated the relative *Bax* and *Bcl-2* transcript levels in cocultured granulosa cells. We found significantly higher *Bax* mRNA expression levels in GC cultured with 0.07% HA compared to controls (*P* < 0.05) and GC cultured with 0.035% HA (*P* < 0.01). The higher *Bax* expression can mean that HA acted as stimulus initiating apoptosis. Although no significant differences were noted in *Bcl-2* relative expression between analysed groups of GC, the highest *Bcl-2* expression was observed in GC cultured with 0.07% HA. These data show a typical interplay between the pro- and antiapoptotic genes and proteins in the apoptotic pathway [21]. The incidence of apoptosis in granulosa cells/cumulus cells is treated as a good indicator of oocyte developmental competence [27–29], as they regulate the maturation of nucleus and cytoplasm in oocytes. Moreover, some authors claim that signs of early atresia in oocytes or cumulus cells translate into better developmental competence [21, 30]. Taking the above mentioned points into account, it may be risky to increase the concentration of HA during IVM above a final concentration of 0.07%. Because the cleavage rate of oocytes cultured with 0.07% HA was the lowest and the *Bax* expression of GC was the highest, these culture conditions may not be optimal for the oocytes, as some are eliminated; however, the remaining oocytes are of better quality, which is reflected in the increased number of developed blastocysts and improved quality (lowest DCI).

In conclusion, our results suggest that addition of 0.07% HA to the *in vitro* maturation medium significantly decreases the level of blastocyst DNA fragmentation. The final concentration of HA being supplemented to oocyte maturation media is critical for the success of the IVP procedure. Moreover, there is a noticeable positive impact on the number of developed blastocysts with 0.07% HA during IVM.

## Figures and Tables

**Table 1:** Effect of HA on oocytes meiotic maturity and chromatin fragmentation (replicates = 4).

Medium	Oocytes *n*	Maturation stage		
		MII *n* (%) (mean ± S.D)	MI *n*	GV *n*
Control	151	106 (70.2%)	42	3
		(26.5 ± 4.36)		

0.035% HA	133	90 (67.7%)	37	6
		(22.5 ± 5.45)		

0.07% HA	144	91 (63.1%)	48	5
		(22.75 ± 5.91)		

Values within column do not differ significantly (test *χ* ^2^).

*n*: number; MII: metaphase II; MI: metaphase I; GV: germinal vesicle.

**Table 2:** The effect of HA supplementation on developmental competence of bovine oocytes (5 replicates) (mean ± S.D.).

Medium	*n* of oocytes	Cleaved *n* (%) (mean ± S.D.)	Blastocyst *n* (%) (mean ± S.D.)
0.035% HA	174	114 (65.5)	22 (13)
		(22.8 ± 5.26)^A,a^	(4.4 ± 3.04)^a^

0.07% HA	169	124 (73.4)	39 (23.1)
		(24.8 ± 7.05)^b^	(7.8 ± 2.38)^b^

Control	186	148 (79.5)	33 (17.7)
		(29.6 ± 6.66)^B^	(6.6 ± 4.97)

Values within column with different superscripts differ significantly: ^a,b^ *P* < 0.05;  ^A,B^ *P* < 0.001 (test *χ* ^2^). *n*: number.

**Table 3:** The effect of HA supplementation on blastocyst quality measured by DNA fragmentation of blastomeres.

Medium	*n* blastocysts	Mean number of nuclei per blastocyst ± S.D.	Mean number of apoptotic nuclei per blastocyst ± S.D.	Mean DCI per blastocyst ± S.D.
0.035% HA	20	84.4 ± 33.82^a^	9.1 ± 7.64^a^	13.91 ± 18.2
0.07% HA	29	86.83 ± 38.95^a^	6.45 ± 3.28^a,A^	8.89 ± 5.35^C^
Control	27	81.67 ± 27.65^a^	14.37 ±7.15^b,B^	20.34 ± 13.5^D^

^a,b^ *P* < 0.05, ^A,B^ *P* < 0.001, ^C,D^ *P* < 0.01 ANOVA followed by Tuckey's post hoc test.

*n*: number; DCI: death cell index.
